# A brief online writing intervention improves positive body image in adults living with dermatological conditions

**DOI:** 10.3389/fmed.2022.1064012

**Published:** 2022-12-21

**Authors:** Kate V. Adkins, Paul G. Overton, Andrew R. Thompson

**Affiliations:** ^1^Department of Psychology, University of Sheffield, Sheffield, United Kingdom; ^2^South Wales Clinical Psychology Training Programme, School of Psychology, Cardiff and Vale University Health Board, Cardiff University, Cardiff, United Kingdom

**Keywords:** psychodermatology, appearance anxiety, body appreciation, functionality appreciation, skin shame

## Abstract

**Introduction:**

Dermatological conditions can affect how individuals feel about their bodies. This research therefore seeks to evaluate the potential for a brief writing intervention, focused on body functionality, to improve body image in adults living with a range of dermatological conditions.

**Methods:**

As part of a parallel Randomised Controlled Trial, 451 adults living with a dermatological condition were randomized to either three functionality-based writing tasks or three creative writing tasks (control). Of these, 155 participants completed pre- and post-intervention measures of body appreciation, functionality appreciation, appearance anxiety, skin-related shame, and skin-related quality-of-life.

**Results:**

For participants with relatively low or mid-range scores on baseline body appreciation and functionality appreciation, there were medium-to-large positive effects of the intervention. Effects were smaller, with all but-one remaining significant, at 1-month follow up and in intention-to-treat analyses. No between-group effects of the intervention were found for measures of appearance anxiety, skin-related shame, and skin-related quality-of-life.

**Discussion:**

These findings suggest that a 1-week writing intervention has the potential to improve positive aspects of body image, but not appearance- and skin-related distress in adults living with a dermatological condition.

**Clinical trial registration:**

[https://clinicaltrials.gov/ct2/history/NCT044459 74?V_3=View], identifier [NCT04445974].

## Introduction

Dermatological conditions include a range of disorders and diseases that affect the functioning of the hair, skin, and/or nails. Existing research has identified the potential wide-ranging impact of skin conditions. In a global burden of disease study, skin diseases collectively accounted for the fourth greatest non-fatal burden of disease, with dermatitis, acne, urticarial, and psoriasis among the most burdensome (1). UK Population health surveys indicate approximately 54% of the adult population have a skin condition (2, 3).

Epidemiological studies report elevated levels of mental health difficulties, including depression, anxiety, and Body Dysmorphic Disorder (BDD) in populations with chronic skin conditions compared to the general population (2). For example, BDD, where individuals experience high levels of preoccupation and distress around a perceived flaw in their appearance, were estimated to have prevalence rates of 11.3% in dermatological populations as opposed to 1.9% in the general population (4).

Given skin is both visible and the body’s largest organ, there is potential for skin conditions to lead to appearance concerns. Visible skin conditions are predominantly defined as conditions that affect the appearance of the skin in areas difficult to cover with clothing, such as the face, neck, and hands (5, 6) and are a leading cause of visible difference (7). It is therefore unsurprising dermatological conditions have the potential to influence how individuals relate to and evaluate their bodies. For example, qualitative and survey studies highlight the challenges skin conditions can pose to aspects of body and skin satisfaction, which are often associated with a desire to conceal the visible signs of the condition and avoid situations where the skin condition may be exposed (8–11). Furthermore, in the qualitative literature, appearance-related concerns have been consistently cited as a central aspect of living with a dermatological condition (10, 12, 13, 14).

Treatments for dermatological conditions primarily focus on physical signs and symptoms. Such treatments and advances play an important role in the management of dermatological conditions and in turn quality-of-life. However, clinician rated-severity correlates poorly with appearance-related distress (15). Instead, psychosocial variables including self-rated severity appear to be stronger predictors of distress (16). While effective medical treatments can improve psychosocial wellbeing, reports from the All Party Parliamentary Group on Skin [APPGS] (2, 17) emphasize the need to increase research and awareness of the impact of living with dermatological conditions and the need to improve both psychological and medical treatment.

Self-help interventions have the potential to provide flexible and discrete access to psychological interventions (18). However, existing evidence for specific self-help interventions targeting body image in adults living with a dermatological condition and/or visible differences is currently limited (19–21). Furthermore, a meta-analysis estimated medium-sized effects of psychological interventions on skin-disease severity, psychosocial measures, and itch-scratch cycles (20). However, reviews highlight limitations of the existing research, including a lack of randomised controlled trials (RCTs), lack of detail in reporting data analyses, and potential mechanisms of effects (19–21). Subsequently, there is a call for research using RCTs to evaluate the effectiveness of theory-driven interventions to improve psychosocial wellbeing (19–21).

One intervention with promising results in improving body image in female populations with high levels of body dissatisfaction and student samples, is the brief writing intervention “Expand Your Horizon” (EYH: 22, 23). Compared to controls, participants completing EYH reported increased levels of body satisfaction (22, 23), body appreciation (22, 23), body functionality (22, 23), body complexity (23), and lower levels of self-objectification (22). Effects were maintained at 1-week (22, 23) and 1-month follow up (23). Findings were replicated in an RCT evaluating the effectiveness of the intervention adapted for a clinical population with rheumatoid arthritis, with the additional finding that depression, but not anxiety, significantly improved in the intervention group (24). Evaluations around the importance of physical appearance are proposed to influence psychosocial adjustment in individuals with dermatological conditions (25, 26). EYH was therefore identified as a potential intervention to target the value placed on appearance and in-turn body image.

Expand Your Horizon can be delivered online and comprises of three writing exercises completed over the course of 1-week, encouraging participants to focus on their body-functionality instead of their physical appearance (22). EYH is based on principles of positive psychology whereby positive body image is not primarily the level of dissatisfaction and/or satisfaction, but is holistic and incorporates acceptance, appreciation of diversity and functionality (27). There is a growing area of research examining body functionality as a modifiable aspect of positive body image. Body functionality encompasses multiple domains, such as internal processes, health, self-care, senses, communication, creativity, and physical activities (28). Alleva et al. (28) argue that by training individuals to shift their focus from appearance to functionality, individuals can develop a more positive relationship with their body. This shift can also be understood with self-objectification theory, which posits that women, in particular, are socialized from an early age to view their bodies “from the outside,” as objects to be looked at (29), and focusing on functionality allows women, including women with disabilities, to develop healthier relationships with their bodies (28, 30).

The primary aim of this study was to test whether, compared to a control condition, a brief functionality writing intervention could improve positive body image in individuals living with dermatological conditions, as measured by body and functionality appreciation. We hypothesized that participants completing the functionality intervention, compared to participants completing a control writing task, would report significantly higher levels of positive body image on post-intervention and follow up measures of functionality and body appreciation.

A secondary aim was to test whether the writing intervention could improve levels of psychological wellbeing on measures of skin-related shame, appearance anxiety, and quality-of-life. We hypothesized that participants completing the functionality intervention, compared with participants completing the control tasks, would report lower levels of appearance anxiety, skin-related shame, and impaired quality-of-life.

## Materials and methods

This study adopted a parallel RCT design to assess the effectiveness of an online brief writing intervention EYH, compared to a control condition, on body image in a population with dermatological conditions. The study protocol was pre-registered on ClinicalTrials.gov. Ethical approval was granted by the University of Sheffield ethics committee (reference number: 032128).

### Participants

Eligible participants were age 18 or above, who self-reported having a dermatological condition that affects their body image. Dermatological conditions include health conditions that affect the hair, skin, and/or nails, but exclude dermatological changes due to traumatic injuries (e.g., burns). Participants were required to have sufficient English to complete the measures and writing exercises. Individuals were excluded if they did not consent to being randomly allocated to the intervention or control condition, completing three writing tasks or participating in the study.

*A priori* power analysis based on ANCOVA (for the primary outcome – body appreciation) was conducted using G*Power (31) to estimate the sample size required to achieve 80% power with a significance level of 0.05. Based on previous RCTs using EYH, a medium-sized effect was assumed [see (32) for a systematic review of positive body image interventions]. Assuming a medium effect size of *f* = 0.25, the total sample size required was 128.

Participants were recruited from a community sample. The study was advertised across various platforms including: University staff and student volunteers lists, social media/forums, charities (e.g., Alopecia UK; British Skin Foundation, Verity UK), and mailing lists of individuals who had previously participated in similar research. A total of 451 participants were randomized to the intervention (*n* = 228) and control (*n* = 223) conditions. Of these, 155 participants (34.4%) provided at least one follow up measure. In the intervention condition, 71 (31.1%) completed a follow up measure 1-month later, whereas within the control condition, 79 (35.4%) participants completed the post-intervention. Dropout was comparable across both conditions, and there were no significant differences in the number of non-completers between the intervention and control conditions [*X*^2^ (1, *N* = 451) = 0.44, *p* = 0.51, φ = 0.03]. Characteristics of participants in the intervention and control conditions are presented in Table 1 and Supplementary Tables 1, 2. Checks indicated that randomization was successful. Intervention and control groups did not significantly differ on key demographic and clinical variables or baseline measures.

**TABLE 1:** Participant demographics.

**Demographics**	**Participant characteristics**	**Intervention (*n* = 228)**	**Control (*n* = 223)**	**Statistics**
Age (years)		*M* = 35.8, *SD* = 12.9, Range = 18–80	*M* = 34, *SD* = 11.1, Range = 18–76.	*t*(*441*) = *1.54, p* = *0.12*
Gender	Female	*n* = 198 (87.6%)	*n* = 195 (88.2%)	*X*^2^ (2, *N* = 447) = 0.047, *p* = 0.98
	Male	*n* = 26 (11.5%)	*n* = 24 (10.9%)	
	Other	*n* = 2 (0.9%)	*n* = *2* (*0.9*)	
Ethnicity	White	*n* = 195 (85.5%)	*n* = 174 (76.3%)	*X*^2^ (5, *N* = 450) = 7.9, *p* = 0.16
	Asian	*n* = 18 (7.9%)	*n* = 26 (11.7%)	
	Mixed	*n* = 10 (4.4%)	*n* = 10 (4.5%)	
	Black	*n* = 5 (2.2%)	*n* = 9 (4.1%)	
	Arab		*n* = 2 (0.9%)	
	Latin American		*n* = 1 (0.5%)	
Paid work?	Yes	*n* = 148 (64.9%)	*n* = 148 (67.9%)	*X*^2^ (1, *N* = 446) = 0.044, *p* = 0.51
Higher education?	Yes	*n* = 156 (65.8%)	*n* = 148 (67.3%)	*X*^2^ (1, *N* = 445) = 0.02, *p* = 0.64

### Intervention

Participants allocated to the intervention received EYH (22). EYH consists of three writing exercises, typically completed over 6-days. Participants were asked to write for 15 mins each time, focusing on specific functions (e.g., functions related to communication and senses) that their body performs and why these functions are important (e.g., enjoyment from listening to music). The self-guided intervention is intended to help individuals practice thinking about what their body does for them, rather than what it looks like or cannot do. The wording of intervention materials, including the introduction and examples were adapted for a mixed-gender population with various dermatological conditions. Three experts-by-experience with different dermatological conditions and different backgrounds piloted the intervention. Their feedback was used to refine the intervention materials before being reviewed by the experts by experience and the author of the intervention.

### Procedure

All components of the study were conducted online *via* Qualtrics (Qualtrics, Provo, UT, USA) to aid the blinding process.

At Timepoint 1 (T1), prospective participants self-identifying as having a dermatological condition that affects their body image were provided with information outlining the inclusion criteria, the broad purpose of the study, and what participation would involve. Participants were asked to confirm whether they had read the information and consented to: (1) participating in the study; (2) completing three 15 min writing tasks over 1-week; (3) being randomized to either the intervention or the control condition. Participants were then asked to complete the demographic measures (gender, age, ethnicity, educational level, and employment status) and provide information on their dermatological condition(s). This included duration, location, diagnosis, visibility and perceived severity. Participants were also asked if they had any other diagnosed health conditions, and whether they were receiving any psychological/pharmaceutical interventions.

Immediately after this, participants completed counterbalanced trait measures relating to body image (body functionality, body appreciation, and appearance anxiety), and skin-related shame and quality-of-life. The online system then randomly allocated participants, at a ratio of 1:1, to either EYH or a sham control (creative writing). Participants were asked to complete the first writing task, before rating their state appearance satisfaction, skin satisfaction, and functionality satisfaction, and providing an email to receive the links to the remaining exercises. Participants were not told whether they had been assigned to the intervention or control condition until the end of the 1-month follow up. Participants could unblind themselves by exiting the study and requesting the debrief information.

Two days later (Timepoint 2, [T2]), participants were sent an automated email with a link to the second writing exercise. Participants were asked to complete the writing exercise, before re-rating the state measures. A further 2 days later (Timepoint 3 [T3]), participants were asked to complete the final writing task and re-rate state measures, before repeating the counterbalanced trait measures given at baseline. One-month after completing the final writing task (Timepoint 4 [T4]), participants received a link to the final set of counterbalanced body image, and skin-related questionnaires, and again were asked to re-rate the state measures.

If participants did not complete part of the study, they received an additional reminder email. Following completion of the questionnaires, participants were shown the debrief information and unblinded. Most participants in the intervention and control groups fully adhered to the writing instructions. All participants regardless of condition were able to download a copy of the intervention materials at the end of the study.

### Trait measures

Information on the measures presented to participants are provided below. Cronbach’s alphas (α) were calculated using survey data to assess the internal consistencies of measures within this study. All scales showed good-to-excellent internal reliability (α ≥ 0.85).

#### Body appreciation

The Body Appreciation Scale-2 (BAS-2: 27) was used to measure trait levels of body appreciation. Each of the 10 items (e.g., “I appreciate the different and unique characteristics of my body”) are rated on a scale of 1 (never) to 5 (always). Average score is calculated by adding each item and dividing by 10. Average scores range between 1 and 5, with higher numbers indicating higher levels of body appreciation. The scale had excellent internal reliability (α = 0.94), and has established construct, concurrent validity, and 3-week test-retest reliability (27). In previous trials of EYH, the BAS-2 has been responsive to change (22, 23).

#### Functionality appreciation

The Functionality Appreciation Scale (FAS: 33), comprising of seven questions, was used to assess participants’ trait levels of appreciation for their bodies’ functionality. Each item (e.g., “I am grateful that my body enables me to engage in activities that I enjoy or find important.”) is rated on a scale from 1 (strongly disagree) to 5 (strongly agree). Average score is calculated by adding each item and dividing by 7. Average scores range between 1 and 5, with higher numbers indicating higher levels of function appreciation. The scale had excellent internal reliability within this study (α = 0.90), and has established construct, concurrent validity, and 3-week test-retest reliability (22). In previous trials of EYH, the FAS has been responsive to change (22, 23).

#### Appearance anxiety

The Appearance Anxiety Index (AAI: 34) was used to measure appearance anxiety. The AAI contains 10 questions (α = 0.86) focused on cognitive and behavioral components of appearance-related anxiety, including avoidance (e.g., “I try to camouflage or alter aspects of my appearance”) and threat monitoring (e.g., “I check my appearance, e.g., in mirrors, by touching with my fingers, or by taking photos of myself”). Each item is scored on a five-point Likert scale from 0 (not at all) to 4 (all the time). Total scores can range from 0 to 40, with higher scores indicating greater levels of appearance-related anxiety. The AAI is responsive to change from interventions and scores of 20 or above indicate clinical levels of appearance anxiety (35).

#### Skin shame

The Skin Shame Scale (SSS: 36) was used to measure levels of skin-specific shame. The SSS contains 24 items (e.g., “I avoid intimate contact because of my skin”), which are rated on a scale from 1 (never) to 5 (always). Total scores can range from 24 to 120, with higher scores indicating greater levels of shame. The SSS had excellent internal consistency in this study (α = 0.90), and has good construct validity (36, 37).

#### Quality-of-life

The Dermatology Quality of Life Index (DLQI: 38) was used to measure the impact of skin-conditions on participants’ quality-of-life. The DLQI contains 10 questions (e.g., “Over the last week how embarrassed or self-conscious have you been because of your skin”) scored on a Likert scale from 0 (not at all/not relevant) to 3 (very much). Total scores range from 0 to 30, with lower scores indicating greater skin-specific quality-of-life. Scores are categorized into “no impact” (0–1), “small impact” (2–5), “moderate impact” (6–10), “very large” (11–20), “extremely large” (21–30) (39). Internal consistency in this study was good (α = *0.85*), and the scale is reported to have good test-retest reliability and construct validity (40). A change in score of 4 or more indicates clinical and reliable change (41).

### State measures

After each writing exercise, participants were asked to rate their state appearance satisfaction, skin-appearance satisfaction and body-functionality satisfaction, on a 100-point visual analogue scale. Visual analogue scales are commonly used in experimental research to measure state changes in body image (42).

### Analytic strategy

Data were analyzed using SPSS v.26 (IBM, Armonk, NY, USA: IBM Corp). Checks for normality using visual inspection (histograms) and absolute measures of skewness and kurtosis indicated outcome measures were approximately normally distributed. Outcome data from the DLQI were non-normally distributed, therefore, independent samples *t*-tests were used to test group differences post-intervention (T3–T1) and at follow up (T4–T1).

To assess whether randomization of allocation to groups (intervention vs. control) was effective, *t*-tests, chi-squared tests and ANOVAs were used, as appropriate, to compare participant characteristics, including demographics, dermatological history, and baseline scores on the outcome measures. To check whether the writing task manipulation was effective, *t*-tests were used to compare state functionality appreciation immediately after each writing task. Between group differences on state measures were compared for each timepoint.

Effectiveness of the intervention was tested in two ways. Firstly, for those participants who completed all stages of the procedure (“completers”), effectiveness was tested using a series of between-group ANCOVAs, with group (functionality vs. control) as the independent variable, post-intervention scores on the BAS-2, FAS, AAI, and SSS as the dependent variables, and baseline scores on the corresponding measure as the covariate. Secondly, for primary outcome measures (BAS-2 and FAS), ANCOVAs were rerun with intention-to-treat (ITT) analyses using the last-observation-carried-forward method for missing data. Initial assumption checks for the ANCOVAs indicated the assumptions of homogeneity of regression slopes may have been violated. Visual inspection of scatter plots indicated the strength of effects of the intervention and control at T3 and T4 may differ at different levels of the covariate (baseline scores). Consequently, interaction terms were included in ANCOVA models. ANCOVAs were run with the corresponding baseline (T1) score as the covariate at three levels: (1) one-standard deviation below the mean; (2) the mean; and (3) one-standard deviation above the mean, to differentiate effects for participants with relatively low, mid-range, and high baseline scores, respectively. Sidak’s correction was used to correct for multiple comparisons.

The number of participants meeting the criteria for clinical change on measures of appearance anxiety and skin-specific quality-of-life were calculated for each group.

## Results

### Manipulation checks/state outcomes

A series of independent samples *t*-tests (Table 2) indicated the participants who completed the functionality tasks scored significantly higher than participants who completed the creativity tasks on state functionality appreciation at T1 [*t*(259) = 4.35, *p* < 0.001, *d* = 0.54], T2 [*t*(164) = 3.77, *p* < 0.001, *d* = 0.59], T3 [*t*(149) = 3.65, *p* < 0.001, *d* = 0.59], as well as at T4, [*t*(142) = 2.91, *p* = 0.004, *d* = 0.49]. There was a small marginally significant difference for skin satisfaction at 1 month follow up [*t*(142) = 2.09, *p* = 0.038, *d* = 035]. However, no other differences were statistically significant.

**TABLE 2:** Mean (SD) scores on state measures immediately following each writing task for participants in the functionality and creative condition.

	Body functionality		Body satisfaction		Skin satisfaction	
	**Functionality**	**Creativity**	**Functionality**	**Creativity**	**Functionality**	**Creativity**
T1	69.4 (22.5)***	56.6 (24.9)	44.1 (27.1)	39.8 (25.7)	33.1 (24.4)	31.3 (23.6)
T2	71.3 (19.7)***	59.1 (22.9)	47.5 (22.9)	46.3 (23.6)	45.7 (23.8)	42.0 (23.5)
T3	73.5 (21.3)***	60.4 (22.8)	52.6 (23.7)	48.8 (24.6)	50.1 (24.1)	46.7 (25.9)
T4	72.9 (21.4)***	61.6 (24.9)	52.2 (22.8)	44.3 (25.0)	48.1 (24.4)*	39.4 (24.8)

*p < 0.05; ***p < 0.005.

### Body appreciation

Results of the ANCOVAs comparing completers post-intervention scores on the BAS-2, indicated there was a positive effect of the intervention on body appreciation (Table 3). Participants completing functionality exercises, as opposed to creativity exercises, reported significantly greater body appreciation post-intervention. Effect sizes were moderate for participants with relatively low [*F*(1,147) = 14.36, *p* ≤ 0.001, η*p*^2^ = 0.089]; and midrange [*F*(1,147) = 17.55, *p* ≤ 0.001, η*p^2^* = 0.11], pre-intervention scores, and small for participants with relatively high initial scores [*F*(1,147) = 4.54, *p* = 0.035, η*p^2^* = 0.030]. At 1-month follow up, the effect of the intervention remained significant, but reduced to small for participants who initially had low [*F*(1,147) = 8.09, *p* = 0.005, η*p^2^* = 0.055], or mid-point [*F*(1,147) = 7.47, *p* = 0.007, η*p^2^* = 0.051], scores on the BAS-2, while between-group differences became non-significant for participants with relatively high initial scores [*F*(1,147) = 0.92, *p* = 0.34, η*p^2^* = 0.007]. However, there were no significant effects of the interaction between baseline BAS-2 score and study arm (intervention vs. control) on post-intervention and follow up BAS-2 scores.

**TABLE 3:** Summary of completer analysis for body appreciation (BAS-2), including estimated marginal means and effects of the intervention at baseline values of BAS-2 one-standard deviation below the mean, the mean, and one-standard deviation above the mean, as well as the interaction effect (baseline BAS-2 and study arm) on BAS-2 post-intervention (*n* = 151) and at 1-month follow up (*n* = 144).

Group	BAS-2 (pre)	BAS-2 (post-intervention)				Effect?		BAS-2 (follow up)				Effect?	
	***M***	***n***	***M***	***SE***	***CI***	***p***	**η*p*^2^**	***n***	***M***	***SE***	***CI***	***p***	**η*p*^2^**
Functionality	2.04	10	2.61	0.069	2.47–2.74	≤0.001	0.089	10	2.63	0.083	2.47–2.80	0.005	0.055
Creativity		14	2.25	0.063	2.13–2.38			13	2.31	0.079	2.15–2.46		
Functionality	2.78	52	3.19	0.048	3.10–3.28	≤0.001	0.11	51	3.18	0.058	3.07–3.30	0.007	0.051
Creativity		55	2.92	0.045	2.83–3.00			52	2.96	0.057	2.85–3.07		
Functionality	3.52	10	3.77	0.067	3.64–3.91	0.035	0.030	10	3.76	0.84	3.6–3.93	0.34	0.007
Creativity		10	3.58	0.065	3.45–3.70			8	3.65	0.083	3.49–3.81		
		Interaction: *F*(1, 147) = 1.36, *p* = *0.25*, η*p^2^* = 0.009						Interaction: *F*(1, 140) = 1.65, *p* = *0.20*, η*p^2^* = 0.012					

In post-intervention ITT analyses (Table 4), participants randomized to functionality exercises, as opposed to creativity exercises, reported significantly greater body appreciation. Effect sizes were medium for participants with relatively low or midrange pre-intervention scores, and small for participants with relatively high scores [low [*F*(1,447) = 5.92, *p* = 0.015, η*p^2^* = 0.013]; mid-range [*F*(1,447) = 11.32, *p* = 0.001, η*p^2^* = 0.025], and high [*F*(1,447) = 5.43, *p* = 0.020, η*p^2^* = 0.012]. However, at 1-month follow up, between-group differences became non-significant for participants with relatively low [*F*(1,147) = 3.27, *p* = 0.071, η*p^2^* = 0.007], and high [*F*(1,447) = 1.32, *p* = 0.252, η*p^2^* = 0.003] pre-intervention scores, but remained significant for participants with midrange scores [*F*(1,147) = 4.35, *p* = 0.038, η*p^2^* = 0.010]. However, there were no significant effects of the interaction between baseline BAS-2 score and condition allocation (intervention versus control) on post-intervention and follow up BAS-2 scores.

**TABLE 4:** Summary of intention-to-treat (ITT) analysis for body appreciation (BAS-2), including estimated marginal means and effects of the intervention at baseline values of BAS-2 one-standard deviation below the mean, the mean, and one-standard deviation above the mean, as well as the interaction effect (baseline BAS-2 and study arm) on BAS-2 at post-intervention and 1-month follow up (*N* = 451).

Group	BAS-2 (pre)	BAS-2 (post-intervention)				Effect?		BAS-2 (follow up)				Effect?	
	***M***	***n***	***M***	***SE***	***CI***	***p***	**η*p*^2^**	***n***	***M***	***SE***	***CI***	***p***	**η*p*^2^**
Functionality	1.84	41	2.00	0.27	1.95–2.05	0.015	0.13	41	2.00	0.031	1.94–2.06	0.071	0.007
Creativity		36	1.91	0.27	1.85–1.96			36	1.92	0.031	1.86–1.98		
Functionality	2.63	155	2.77	0.019	2.73–2.81	0.001	0.025	155	2.76	0.022	2.72–2.81	0.038	0.010
Creativity		157	2.68	0.019	2.64–2.72			157	2.70	0.022	2.66–2.74		
Functionality	3.42	32	3.54	0.026	3.48–3.59	0.020	0.012	32	3.52	0.030	3.46–3.58	0.252	0.003
Creativity		30	3.48	0.27	3.39–3.50			30	3.47	0.031	3.41–3.53		
		Interaction: *F*(1, 447) = 0.006, *p* = *0.94*, η*p^2^* ≤ 0.001						Interaction: *F*(1, 447) = 0.22, *p* = *0.64*, η*p^2^* ≤ 0.001					

### Functionality appreciation

Results of the ANCOVAs comparing participant’s post-intervention scores on the FAS (Table 5), indicated there was an effect of the intervention on functionality appreciation, moderated by completers’ baseline FAS scores. Post-intervention, participants in the intervention condition who started with low [*F*(1,147) = 27.3, *p* < 0.001, η*p*^2^ = 0.16] or mid-range [*F*(1,147) = 23.44, *p* < 0.001], η*p^2^* = 0.14] scores on the FAS scored significantly higher than participants with similar scores within the control group. However, for participants with initially high scores, between-group differences were non-significant [*F*(1,147) = 2.74, *p* = 0.10, η*p^2^* = 0.018]. At 1-month follow up, between-group differences for initially low [*F*(1,139) = 12.9, *p* < 0.001, η*p^2^* = *0.085*], and mid-range [*F*(1,139) = 10.0, *p* = 0.002, η*p*^2^ = 0.067] scorers remained significant, but effect sizes reduced from large to medium. Differences remained non-significant for relatively high scorers [*F*(1,139) = 0.74, *p* = 0.39, η*p^2^* = 0.005]. There was a small but significant interaction of baseline FAS scores and condition (intervention vs. control) on post-intervention FAS scores. However, the interaction between baseline FAS score and study arm (intervention vs. control) on follow up FAS score was small and marginally non-significant.

**TABLE 5:** Summary of completer analysis for functionality appreciation (FAS), including estimated marginal means and effects of the intervention at baseline values of FAS one-standard deviation below the mean, the mean, and one-standard deviation above the mean, as well as the interaction effect (baseline FAS and study arm) on FAS at post-intervention (*n* = 151) and 1-month follow up (*n* = 143).

Group	FAS (pre)		FAS (post-intervention)			Effect?			FAS (follow up)			Effect?	
	***M***	***n***	***M***	***SE***	***CI***	***p***	**η*p*^2^**	***n***	***M***	***SE***	***CI***	***p***	**η*p*^2^**
Functionality	2.93	9	3.77	0.091	3.59–3.95	≤0.001	0.16	8	3.82	0.100	3.62–4.02	≤0.001	0.085
Creativity		13	3.14	0.078	2.98–3.29			11	3.34	0.091	3.16–3.15		
Functionality	3.70	52	4.24	0.55	4.13–4.35	≤0.001	0.14	52	4.16	0.067	4.03–4.30	0.002	0.067
Creativity		51	3.87	0.53	3.77–3.97			47	3.86	0.067	3.74–3.99		
Functionality	4.47	11	4.60	0.074	4.45–4.74	0.10	0.018	11	4.51	0.095	4.32–4.69	0.39	0.005
Creativity		15	4.43	0.072	4.28–4.57			14	4.39	0.095	4.2–4.58		
		Interaction: *F*(1, 147) = 7.94, *p* = *0.006*, η*p^2^* = 0.051						Interaction: *F*(1, 139) = 3.75, *p* = *0.055*, η*p^2^* = 0.026					

Within ITT analyses (Table 6), effects of the intervention on functionality appreciation were significant, but small for participants with low baseline scores at T3 [*F*(1,447) = 9.22, *p* = 0.003, η*p^2^* = 0.020], and at follow up (T4) [*F*(1,447) = 4.12, *p* = 0.043, η*p^2^* = 0.009], and participants with mid-range baseline scores at T3 [*F*(1,447) = 11.62, *p* = 0.001, η*p^2^* = 0.025] and at follow up (T4) [*F*(1,447) = 5.35, *p* = 0.021, η*p^2^* = 0.012]. For relatively high baseline scorers on the FAS, there were no significant effects of intervention allocation on functionality appreciation at T3 [*F*(1,447) = 3.22, *p* = 0.074, η*p^2^* = 0.007], or at follow up (T4) [*F*(1,447) = 1.55, *p* = 0.214, η*p^2^* = 0.003]. In ITT analyses, there were no significant effects of the interaction between baseline FAS score and condition (intervention versus control) on post-intervention and follow up FAS scores.

**TABLE 6:** Summary of intention-to-treat (ITT) analysis for functionality appreciation (FAS), including estimated marginal means and effects of the intervention at baseline values of FAS: one-standard deviation below the mean, the mean, and one-standard deviation above the mean, as well as the interaction effect (baseline FAS and study arm) on FAS at post-intervention and 1-month follow up (*N* = 451).

Group	FAS (pre)		FAS (post-intervention)			Effect?		FAS (follow up)				Effect?	
	***M***	***n***	***M***	***SE***	***CI***	***p***	**η*p*^2^**	***n***	***M***	***SE***	***CI***	***p***	**η*p*^2^**
Functionality	2.76	34	3.02	0.035	2.95–3.09	0.003	0.020	34	3.00	0.038	2.92–3.07	0.043	0.009
Creativity		41	2.86	0.033	2.81–2.94			41	2.89	0.36	2.82–2.96		
Functionality	3.58	157	3.76	0.024	3.71–3.80	0.001	0.025	157	3.72	0.026	3.67–3.77	0.021	0.012
Creativity		144	3.64	0.024	3.59–3.69			144	3.64	0.026	3.58–3.69		
Functionality	4.40	37	4.50	0.043	4.43–4.56	0.074	0.007	37	4.45	0.037	4.38–4.52	0.214	0.003
Creativity		38	4.41	0.035	4.34–4.48			38	4.38	0.038	4.31–4.46		
		Interaction: *F*(1, 447) = 0.78, *p* = *0.38*, η*p^2^* = 0.002						Interaction: *F*(1, 447) = 0.31, *p* = *0.58*, η*p^2^* = 0.001					

### Skin shame and appearance anxiety

Results of the ANCOVAs (Table 7) comparing completers’ post-intervention scores on the AAI indicated there were no significant effects of the intervention regardless of whether participants had low [*F*(1,147) = 0.86, *p* = 0.36, η*p^2^* = 0.006]; mid-range, [*F*(1,147) = 1.76, *p* = 0.19, η*p^2^* = 0.012]; or high [*F*(1,147) = 0.88, *p* = 0.35, η*p^2^* = 0.006], baseline scores at T3. Similarly, at follow up (T4) there were no significant effects for participants with low, [*F*(1,140) = 4.12, *p* = 0.054, η*p^2^* = 0.029]; mid-range [*F*(1,140) = 3.11, *p* = 0.080, η*p^2^* = 0.022]; and high [*F*(1,140) = 0.88, *p* = 0.35, η*p^2^* = 0.006], scores on the AAI. Furthermore, there were no significant effects of the interaction between baseline AAI score and condition (intervention vs. control) on post-intervention and follow up AAI scores.

**TABLE 7:** Summary of completer analysis for appearance anxiety (AAI), including estimated marginal means and effects of the intervention at baseline values of AAI one-standard deviation below the mean, the mean, and one-standard deviation above the mean, as well as the interaction effect (baseline AAI and study arm) on AAI at post-intervention (*n* = 151) and 1-month follow up (*n* = 144).

Group	AAI (pre)		AAI (post-intervention)			Effect?			AAI (follow up)			Effect?	
	***M***	***n***	***M***	***SE***	***CI***	***p***	**η*p*^2^**	***n***	***M***	***SE***	***CI***	***p***	**η*p*^2^**
Functionality	11.9	11	10.0	0.96	8.1–11.9	0.36	0.006	10	9.1	1.09	7.0–11.3	0.054	0.029
Creativity		11	11.8	0.89	9.4–12.9			10	12.2	1.03	10.1–14.2		
Functionality	20.2	52	15.5	0.67	14.2–16.8	0.19	0.012	52	14.9	0.76	13.4–15.3	0.080	0.022
Creativity		52	16.7	0.64	15.5–18.0			50	16.8	0.75	15.3–18.3		
Functionality	28.5	9	21.1	1.92	19.1–23.2	0.35	0.006	9	21.1	1.15	18.8–23.4	0.68	0.001
Creativity		16	22.4	0.87	20.7–24.1			13	21.7	1.05	16.7–23.8		
		Interaction: *F*(1, 147) <0.001, *p* = *0.99*, η*p*^2^ <0.001						Interaction: *F*(1, 140) = 1.21, *p* = *0.27*, η*p^2^* = 0.009					

Similarly, results of the ANCOVA (Table 8) comparing completers’ post-intervention scores on the SSS indicated there were no significant effects of the intervention regardless of whether participants had low [*F*(*1,147*) = *1.50, p* = *0.22*,η*p^2^* = *0.010*]; mid-range, [*F*(1,147) = 2.83, *p* = 0.095, η*p^2^* = 0.019]; or high [*F*(1,147) = 1.29, *p* = 0.26, η*p^2^* = 0.009], baseline scores at T3. Furthermore, at follow up (T4) there were no significant effects for participants with low, [*F*(1,139) = 0.59, *p* = 0.45, η*p^2^* = 0.004]; mid-range [*F*(1,139) = 3.13, *p* = 0.079, η*p^2^* = 0.022]; and high [*F*(1,139) = 2.96, *p* = 0.087, η*p^2^* = 0.021], scores on the SSS. Furthermore, there were no significant effects of the interaction between baseline SSS score and condition (intervention versus control) on post-intervention and follow up SSS scores.

**TABLE 8:** Summary of completer analysis for skin shame (SSS), including estimated marginal means and effects of the intervention at baseline values of SSS one-standard deviation below the mean, the mean, and one-standard deviation above the mean, as well as the interaction effect (baseline SSS and study arm) on SSS at post-intervention (*n* = 151) and 1-month follow up (*n* = 143).

Group	SSS (pre)	SSS (post-intervention)				Effect?		SSS (follow up)				Effect?	
	***M***	***n***	***M***	***SE***	***CI***	***p***	**η*p*^2^**	***n***	***M***	***SE***	***CI***	***p***	**η*p*^2^**
Functionality	66.5	11	61.8	1.54	58.8–64.9	0.22	0.01	11	60.97	1.80	57.4–64.5	0.45	0.004
Creativity		13	64.3	1.35	61.6–66.9			10	62.87	1.70	59.5–66.2		
Functionality	80.3	50	73.4	1.03	71.3–75.4	0.095	0.019	48	72.0	1.22	69.6–74.4	0.079	0.022
Creativity		50	75.8	0.99	73.8–77.7			47	75.1	1.21	72.7–77.5		
Functionality	94.1	11	84.9	1.48	82.0–87.8	0.26	0.009	11	82.5	1.74	79.0–85.9	0.087	0.021
Creativity		16	87.2	1.39	84.5–89.9			16	86.6	1.66	83.3–89.9		
		Interaction: *F*(1, 147) = 0.005, *p* = *0.95*, η*p^2^* <0.001						Interaction: *F*(1, 139) = 0.42, *p* = *0.52*, η*p^2^* = 0.003					

### Clinical change

Among completers, 67.7% of participants scored above the threshold (39) for moderately impaired dermatology-related quality-of-life, measured with the DLQI, and 91.6% reported at least some impairment to their quality-of-life. Participants’ changes in DLQI scores post-intervention (T3–T1) ranged between −7 and 12 (*M* = 2.17, *SD* = 4.15) for participants completing functionality exercises and −10 and 10 (*M* = 1.38, *SD* = 3.68) for participants completing creativity tasks, and did not significantly differ between groups [*t*(149) = 1.24, *p* = 0.22, *d* = 0.20]. Similarly, at follow up (T4-T1), changes in DLQI ranged from −8 to 21 (*M* = 2.42, *SD* = 4.15) and −11 to 14 (*M* = 1.23, *SD* = 4.71), and did not significantly differ between groups [*t*(149) = 1.51, *p* = 0.13, *d* = 0.25]. A change of 4 or more indicates clinical and reliable change (41), and at T3, 24 (33.3%) participants in the intervention condition and 20 (25.3%) participants in the control condition showed clinical and reliable improvement, and 7 (9.72%) and 7 (8.86%) showed clinical and reliable deterioration, which did not differ significantly between groups [*X*^2^ (2, *N* = 151) = 1.34, *p* = 0.51, *V* = *0.094*]. At 1-month follow up (T4), 27 (38.0%) participants in the intervention condition and 21 (28.4%) participants in the control condition showed clinical and reliable improvement, and 6 (8.45%) and 10 (13.5%) showed clinical and reliable deterioration, respectively, which did not differ significantly between groups [*X*^2^ (*2, N* = *145*) = *1.34, p* = *0.51, V* = *0.094*].

The clinical threshold for AAI is 20 or above (35). Among completers, 58.1% of participants met the clinical threshold for appearance anxiety. At T3 similar numbers of participants exhibited clinical change in the intervention condition (*n* = 20, 28.2%) and control condition (*n* = 21, 26.6%), with one (1.4%) and two (2.5%) participants exhibiting clinical deterioration, respectively [*X*^2^ (*2, N* = *150*) = *0.27, p* = *0.87, V* = *0.043*]. At follow up (T4), differences between-groups remained non-significant [*X*^2^ (*2, N* = *144*) = *0.78, p* = *0.68, V* = *0.074*] with 21 (29.6%) and two (2.81%) participants in the intervention condition and 19 (25.6%) and 4 (5.41) in the control condition meeting the criteria for clinical improvement and deterioration, respectively.

Clinical cut-offs are not available for the BAS-2, FAS, and SSS, therefore clinical change was not calculated on these measures.

## Discussion

This study examined whether a 1-week body functionality writing intervention could improve body image and reduce appearance/skin-related distress in adults living with a range of dermatological conditions. For this purpose, the potential effectiveness of an adapted version of EYH was examined in a parallel RCT. In line with the primary hypothesis, participants in the intervention condition, as opposed to the control condition, with lower or mid-range baseline levels of body appreciation and functionality appreciation, reported significantly higher levels of positive body image after completing the final exercise and 1-month later. However, effect sizes reduced from medium to small for body appreciation, and large to medium for functionality appreciation. Outcomes remained fairly similar in ITT analyses, although effects of the intervention on body appreciation were small regardless of baseline score, and at follow up the effect only remained significant for participants with mid-range baseline scores. Similarly, ITT analysis indicated that the effect of the intervention on functionality appreciation dropped from large to small at post-intervention, and medium to small at follow up. There remained no effect of the intervention on functionality appreciation for relatively high baseline scorers.

There was evidence that baseline scores on the FAS and the BAS-2 moderated the effect of the intervention on post-intervention functionality appreciation and body appreciation. The moderation indicated that the intervention may be less relevant for individuals with already high levels of functionality appreciation and body appreciation. This may have been a result of a ceiling effect, and the measures were not sensitive enough to detect change in individuals with higher baseline levels of positive body image. That may in fact be the case for functionality appreciation, where the mean pre-intervention score in the high group was 4.47 (for completers)/4.40 (for the ITT analysis) (Tables 5, 6), close to the maximum mean score of 5 on the FAS. However, mean pre-intervention body appreciation scores in the high group – 3.52 for completers and 3.42 for the ITT analysis – were some distance away from the maximum mean score of 5 on the BAS-2. Indeed, in our study, overall completers reported lower levels of baseline body appreciation (intervention: *M* = 2.65, *SD* = 0.79; control: *M* = 2.62, *SD* = 0.80; Supplementary Table 2) compared to participants included in the development of the BAS-2, which used student (*M* = 3.47–3.97, *SD* = 0.73) and community samples (*M* = 3.22–3.47, *SD* = 0.86–0.96: 27).

Another possibility is that the moderation effect reflects the recruitment of participants with higher levels of distress. In a meta-analysis of standalone body image interventions, selection of participants with elevated appearance distress was identified as a moderator (43). In our sample, there appeared to be elevated levels of skin shame, given that the mean baseline score on the SSS (intervention: *M* = 83.2, *SD* = 0.14; control: *M* = 83.0, *SD* = 13.5; Supplementary Table 2) was higher than that reported in the community dermatology sample (*M* = 66.9, *SD* = 17.8) included in the development of the SSS (36). Likewise, in our sample, there appeared to be elevated levels of appearance anxiety, given that the mean baseline score in the AAI (intervention; *M* = 22.0, *SD* = 8.1; control: *M* = 22.0, *SD* = 8.0) was higher than that previously reported elsewhere (*M* = 12.49, *SD* = 8.46; 44), including a community sample with high levels of appearance concern (Median = 13.0, Inter quartile range = 13.5; 34).

Over a third of participants completing the intervention met the criteria for clinical change (39) on the DLQI and close to 30% met the threshold for clinical change (35) on appearance anxiety. However, differences between groups were non-significant. Comparisons of participants’ scores on secondary measures of distress did not support the hypothesis that participants in the intervention would report lower levels of skin shame, appearance anxiety and impaired quality-of-life compared to participants in the control condition. It is unclear why participants did not exhibit improvements on negative aspects of body image and dermatology-related impairments, especially given the high level of impairment found in our sample (see above). It is possible that participants’ scores were influenced by the Coronavirus pandemic. For example, some participants fed back that they felt less self-conscious of their skin due to leaving the house less and face masks concealing their skin. It is also possible that some participants may have felt more self-conscious given facemasks have been known to exacerbate skin conditions (45). Another potential explanation for the difference is that negative and positive body image are separate constructs (46), therefore it is possible that aspects of positive body image are more responsive to change. Consequently, the short nature of the intervention may have been insufficient to reduce feelings of shame or improve quality-of-life, particularly where individuals have experienced intrusive reactions from others. Additionally, the absence of components directly addressing shame and other maintaining factors in appearance and health-related distress may explain the lack of effect, which warrants further investigation. For example, compassion-focused and societal-level approaches have some evidence for reducing shame (47, 48). Whilst our findings do not support the use of EYH to specifically reduce distress associated with living with a dermatological condition, our findings suggest that in a community sample, completion of the intervention does enhance positive body image.

A major limitation of this study is the high rate of attrition (>65%). Attrition is often high in studies testing self-help interventions within populations with visible differences (49–51), as well as in the wider literature on self-help (52, 53), with pure self-help interventions typically reporting lower rates of attrition when compared to wait-list controls and facilitated interventions (50, 54). However, attrition in our study was higher than attrition reported in previous trials of EYH. It is likely aspects of recruitment partly explained this difference. For example, financial incentives and human facilitated enrollment, as used in previous trials, are linked to higher levels of attrition (52). In addition, technical issues in the study likely contributed to the high attrition (e.g., some participants had difficulty loading the writing task, and there were problems with downloading the functions list). In future it may be helpful to offer individuals the option to download the full intervention materials or receive a print copy of the intervention.

In previous research, authors have emphasized the likelihood that participants completing trials of non-facilitated psychosocial interventions are likely to be non-random (55). For instance, participants experiencing positive outcomes and participants higher in motivation are more likely to complete the intervention (55). In order to address high attrition, we employed a conservative method of last-observation-carried-forward to examine the effect of participant assignment on potential outcomes. However, last-observation-carried-forward is associated with increased risk of type two errors (56). Nonetheless, the effect of the intervention remained predominantly significant, though smaller, in conservative ITT analysis, indicating that effects of the intervention on positive body image were relatively robust. Furthermore, high dropout is likely a naturalistic reflection of who will use and potentially benefit from self-help interventions. Future research using writing interventions would benefit from further investigating the reasons for discontinuation as well as examining techniques to retain engagement.

Another important limitation of this study was the relatively short (1 month) follow up period following completion of the intervention. Whilst the follow up provided evidence that there were continued, yet smaller, effects of the intervention on positive body image, it is not possible to conclude whether an effect would be maintained over a longer period. Therefore, future research would benefit from a longer follow up period, such as 6–12 months, with consideration for monitoring and controlling for the effect of changes in severity and/flare ups.

A strength of this study was the use of a “sham” control to differentiate the effect of the functionality writing intervention, beyond writing more generally. Studies using active controls arguably have more robust findings (43). Although not a focus of this study, it is possible that the process of writing creatively had a therapeutic benefit for some participants, given the clinical change detected in the control condition. As with previous studies comparing EYH to matched creative writing tasks (22), there were effects over time for participants allocated to both conditions. This effect may reflect natural changes over time, or active components of the control condition, like distraction and enjoyment. It is possible participants’ emotional responses to the writing tasks may have influenced participants’ subsequent scores on outcome measures.

The findings from this study add further support to the growing evidence that completing a 1-week functionality intervention has the potential to improve functionality appreciation and body appreciation for a range of groups including adults with dermatological conditions, women with rheumatoid arthritis (24), student populations (28, 57), and women with high levels of body dissatisfaction (22). Furthermore, given the brief and low-cost nature of the intervention, it is promising that the effect of the intervention remained at 1-month post-intervention. However, no existing studies have examined the longevity of the intervention beyond 1-month and subsequently further research including longer follow up periods is required.

## Conclusion

This research adopted a RCT design to examine the effectiveness of a 1-week writing intervention on positive body image and skin/appearance-related distress, in a community sample of adults living with a range of dermatological conditions. For participants who did not start the study with relatively high levels of positive body image, there were medium-to-large effects of completing the functionality tasks on body and functionality appreciation, which were generally maintained at 1-month follow up, with small-to-medium effects. However, attrition was high and there were no effects of the intervention, compared to a control, on measures of appearance anxiety, skin-related shame, or quality-of-life.

## Data availability statement

All relevant data is contained within the article. Further inquiries can be directed to the corresponding author.

## Ethics statement

The studies involving human participants were reviewed and approved by Department of Psychology, University of Sheffield. The patients/participants provided their written informed consent to participate in this study.

## Author contributions

KA conducted this study as part of completion of a doctorate in clinical psychology, collected all the data, conducted the analysis, and prepared the first draft of the manuscript. AT and PO supervised the dissertation project at every stage and contributed to revising the manuscript for publication. All authors contributed to the article and approved the submitted version.
