# Supplementary material for: A brief online writing intervention improves positive body image in adults living with dermatological conditions
Source: Front Med (Lausanne). 2022 Dec 21;9:1064012. doi: 10.3389/fmed.2022.1064012 (PMC9810805; doi:10.3389/fmed.2022.1064012)
Supplement: Supplementary file 1 [file Table_1.pdf]

Table S1: Participant dermatological history.

Health	Intervention (*n* = 228)	Control (*n* = 223)	Statistics
Severity	*M* = 3.2, *SD* = 1.1	*M = 3.2, SD = 1.1*	*t(449) = -0.12, p = .87*
Duration (months)	*M* = 221.38 (18.4 years) *SD* = 165, *Range* = 7 months – 71.2 years	*M = 227.6 (19 years), SD = 152, Range = 2 months-60 years*	*t(445) = -0.41, p = .68*
Diagnosis?	*n* = 212 (93%)	*n* = 209 (94.1%)	$X^2(1, N = 450) = .25, p = .62$
Visible?	*n* = 213 (93.4%)	*n = 204, (91.9%)*	$X^2(1, N = 450) = 0.39, p = .53$
Primary skin condition			
Acne	*n* = 28 (12.3%)	*n* = 35 (15.7%)	$X^2(9, N = 451) = 9.4, p = .40$
Psoriasis	*n* = 22 (9.6%)	*n* = 21 (9.4%)	
Eczema	*n* = 63 (27.6%)	*n* = 71 (31.8%)	
Alopecia	*n* = 17 (7.5%)	*n* = 15 (6.7%)	
Vitiligo	*n* = 23 (10.1%)	*n* = 17 (7.6%)	
Rosacea	*n* = 10 (4.4%)	*n* = 3 (1.4%)	
HS	*n* = 9 (3.9%)	*n* = 14 (6.3%)	
Hirsutism	*n* = 23 (10.1%)	*n* = 25 (11.2%)	
Multiple	*n* = 14 (6.1%)	*n* = 9 (4%)	
Other	*n* = 19 (8.3%)	*n* = 13 (5.8%)	
Talking therapy	*n* = 19 (8.3%)	*n* = 24 (10.8%)	$X^2(1, N = 451) = 0.77, p = .38$

Health	Intervention (*n* = 228)	Control (*n* = 223)	Statistics
Psychotropic medication	*n* = 13 (10.6%)	*n* = 8 (3.6%)	X^2*(1, N* = 451*) = 1.14, p* = .29

Table S2. Summary of baseline scores on outcome measures for participants in both conditions.

Measure	Intervention (*n* = 228)	Control (*n* = 223)	Statistics
BAS-2	*M* = 2.65, *SD* = 0.79	*M* = 2.62, *SD* = 0.80	*t*(449) = 0.38, *p* = .70
FAS	*M* = 3.55, *SD* = 0.80	*M* = 3.50, *SD* = 0.90	*t*(449) = 0.90, *p* = .37
AAI	*M* = 22.0, *SD* = 8.1	*M* = 22.0, *SD* = 8.0	*t*(449) = 0.12, *p* = .90
SSS	*M* = 83.2, *SD* = .14	*M* = 83.0, *SD* = 13.5	*t*(449) = 0.17, *p* = .87
DLQI	*Mdn* = 10; *IQR*: 13	Mdn = 11; IQR: 10	*U* = 25102.5, *p* = .94
